# Psychological status and behavior changes of the public during the COVID-19 epidemic in China

**DOI:** 10.1186/s40249-020-00678-3

**Published:** 2020-05-29

**Authors:** Xi Liu, Wen-Tao Luo, Ying Li, Chun-Na Li, Zhong-Si Hong, Hui-Li Chen, Fei Xiao, Jin-Yu Xia

**Affiliations:** 1 Department of Infectious Diseases, The Fifth Affiliated Hospital, Sun Yat-sen University, Zhuhai, China; 2 Guangdong Provincial Engineering Research Center of Molecular Imaging, The Fifth Affiliated Hospital, Sun Yat-sen University, Zhuhai, China; 3 Department of Nursing, The Fifth Affiliated Hospital, Sun Yat-sen University, Zhuhai, China

**Keywords:** COVID-19, Public psychological status, Psychological stress, Behavior changes, Anxiety, Depression, Phobia

## Abstract

**Background:**

A cluster of pneumonia cases were reported by Wuhan Municipal Health Commission, China in December 2019. A novel coronavirus was eventually identified, and became the COVID-19 epidemic that affected public health and life. We investigated the psychological status and behavior changes of the general public in China from January 30 to February 3, 2020.

**Methods:**

Respondents were recruited via social media (WeChat) and completed an online questionnaire. We used the State-Trait Anxiety Inventory, Self-rating Depression Scale, and Symptom Checklist-90 to evaluate psychological status. We also investigated respondents’ behavior changes. Quantitative data were analyzed by *t*-tests or analysis of variance, and classified data were analyzed with chi-square tests.

**Results:**

In total, 608 valid questionnaires were obtained. More respondents had state anxiety than trait anxiety (15.8% vs 4.0%). Depression was found among 27.1% of respondents and 7.7% had psychological abnormalities. About 10.1% of respondents suffered from phobia. Our analysis of the relationship between subgroup characteristics and psychological status showed that age, gender, knowledge about COVID-19, degree of worry about epidemiological infection, and confidence about overcoming the outbreak significantly influenced psychological status. Around 93.3% of respondents avoided going to public places and almost all respondents reduced Spring Festival-related activities. At least 70.9% of respondents chose to take three or more preventive measures to avoid infection. The three most commonly used prevention measures were making fewer trips outside and avoiding contact (98.0%), wearing a mask (83.7%), and hand hygiene (82.4%).

**Conclusions:**

We need to pay more attention to public psychological stress, especially among young people, as they are likely to experience anxiety, depression, and psychological abnormalities. Different psychological interventions could be formulated according to the psychological characteristics of different gender and age groups. The majority of respondents followed specific behaviors required by the authorities, but it will take time to observe the effects of these behaviors on the epidemic.

## Background

Acute respiratory infectious diseases have emerged continuously over the past 20 years. In 2003, a severe acute respiratory syndrome (SARS) epidemic broke out in Guangdong Province, China, which had a lasting impact on public health in China and worldwide [1]. Since then, new epidemic outbreaks have continued to emerge, such as the H5N1 avian influenza A in 2004 [2], the H1N1 influenza A in 2009 [3], the Ebola virus in 2014 [4], and the Middle East respiratory syndrome in 2012 [5]. In December 2019, a series of pneumonia cases without certain etiology occurred in Wuhan, Hubei province of China. The clinical manifestations were similar to viral pneumonia; a new coronavirus was subsequently identified [6]. This disease was later named “coronavirus disease 2019” (COVID-19) by the World Health Organization (WHO) [7]. As of February 8, 2020, 37 539 confirmed COVID-19 cases had been reported worldwide, of which 37 251 were in China; there were 812 deaths [8]. The epidemic situation in China was serious.

On January 30, 2020, the WHO declared the COVID-19 epidemic constituted a public health emergency of international concern [9]. At the same time, almost all provinces or regions in China had initiated Level I responses to public health emergencies. The Chinese central and local government rapidly implemented rigorous measures to control the development of the epidemic, including extending the Spring Festival holiday, canceling large-scale performances, and encouraging the wearing of masks in public places. As the largest epidemic area, the entire city of Wuhan was “on lockdown”.

Individual and collective behavior is particularly important during a pandemic. In the absence of appropriate pharmacological interventions, the main method of controlling outbreaks is to change public behavior. An individual’s behavior can affect their family, social networks, organizations in which they participate, communities to which they belong, information they obtain, and the impact on their society [10]. When people learn about disease information, they usually have an emotional response that affects any immediate behavioral changes. A previous study used mathematical models to show that epidemics can affect individuals’ fears, and that individuals’ emotions may in turn affect behaviors during epidemics [11]. Previous experience suggests that the public is likely to experience anxiety, depression, and panic attacks when faced with highly contagious diseases. A study focused on the avian influenza in France (*n* = 600) reported that 39.0% of participants expressed anxiety about the disease, and 20.0% that had knowledge about avian influenza had changed their behaviors during the epidemic [12]. During the SARS epidemic, a study from Toronto found a high incidence of psychological distress among 129 quarantined individuals. Symptoms of post-traumatic stress disorder and depression were found in 28.9 and 31.2% of respondents, respectively [13]. During the initial stage of the COVID-19 outbreak, 53.8% of Chinese respondents rated the psychological impact of the outbreak as moderate or severe, 16.5% reported moderate to severe depressive symptoms, 28.8% reported moderate to severe anxiety symptoms, and 8.1% reported moderate to severe stress levels [14].

Similar to individual behavior, individual emotions can easily affect collective emotions. Information about the disease, the psychology of the population, and individuals’ behavior interact to influence the spread of an epidemic. Interventions based on these interacting factors can be used to control an epidemic and improve public health. An effective planning and response strategy must consider these complex interactions. However, available studies on COVID-19 have focused on understanding the disease [6, 15–17], epidemiology [17, 18], treatment [19, 20], and vaccines. However, this outbreak highlighted the fragility of psychological resilience, and we also need to pay attention to the psychological status of ordinary people during an epidemic [21]. At the time of this study, the epidemic curve suggested China was approaching the peak of the epidemic. At this critical point, we investigated the psychological status and behavior changes among ordinary Chinese people during the COVID-19 epidemic, and evaluated whether these factors were related to the spread of the disease.

## Methods

### Setting and participants

Because of the outbreak, the Chinese government advised the public to reduce face-to-face interactions and isolate themselves at home. Therefore, we chose to conduct this study through an electronic network survey. We designed a cross-sectional study to investigate the psychological status of the general public in China during the COVID-19 epidemic using an anonymous online questionnaire. The questionnaire was distributed via an online survey platform (Wenjuanxing, www.wjx.cn), and the questionnaire link was sent to respondents through social media (WeChat, Tencent, Shenzhen, China). The survey was conducted from January 30 to February 3, 2020. Respondents were selected by snowball sampling. The questionnaire link was first sent to the family members of hospital employees (non-medical workers); these respondents were then encouraged to forward the link to other family members, friends, and colleagues.

### Questionnaire content

The first part of the questionnaire (see supplementary material) covered general demographic information, including gender, age, and region. The second part included questions developed by the present researchers, such as respondents’ epidemiological history, their understanding of COVID-19, and the impact of the epidemic outbreak. The third part of the questionnaire comprised the State-Trait Anxiety Inventory (STAI, score range 20–80) designed by Spielberger [22] and the Self-rating Depression Scale (SDS) developed by Zung [23], which were used to evaluate respondents’ anxiety and depression, respectively. The final part included the Symptom Checklist-90 (SCL-90) designed by Derogatis [24], which is used to screen for psychological problems other than anxiety and depression.

### Quality control method

We set strict parameters that each social media account was only allowed to answer the questionnaire once, and use of the same Internet protocol address to answer another questionnaire was forbidden to ensure the authenticity of responses. The STAI, SDS, and SCL-90 have all been previously validated and used in Chinese populations [25–27]. Therefore, they were appropriate for use in this study.

### Data analysis

The collected data were analyzed using SPSS version 21.0 (IBM SPSS Statistics, New York, United States). Quantitative data were analyzed by *t-*tests or analysis of variance, and classified data were analyzed by chi-square tests. *P* < 0.05 was considered statistically significant.

## Results

### Respondents’ demographic characteristics and scale scores

**Fig. 1:**
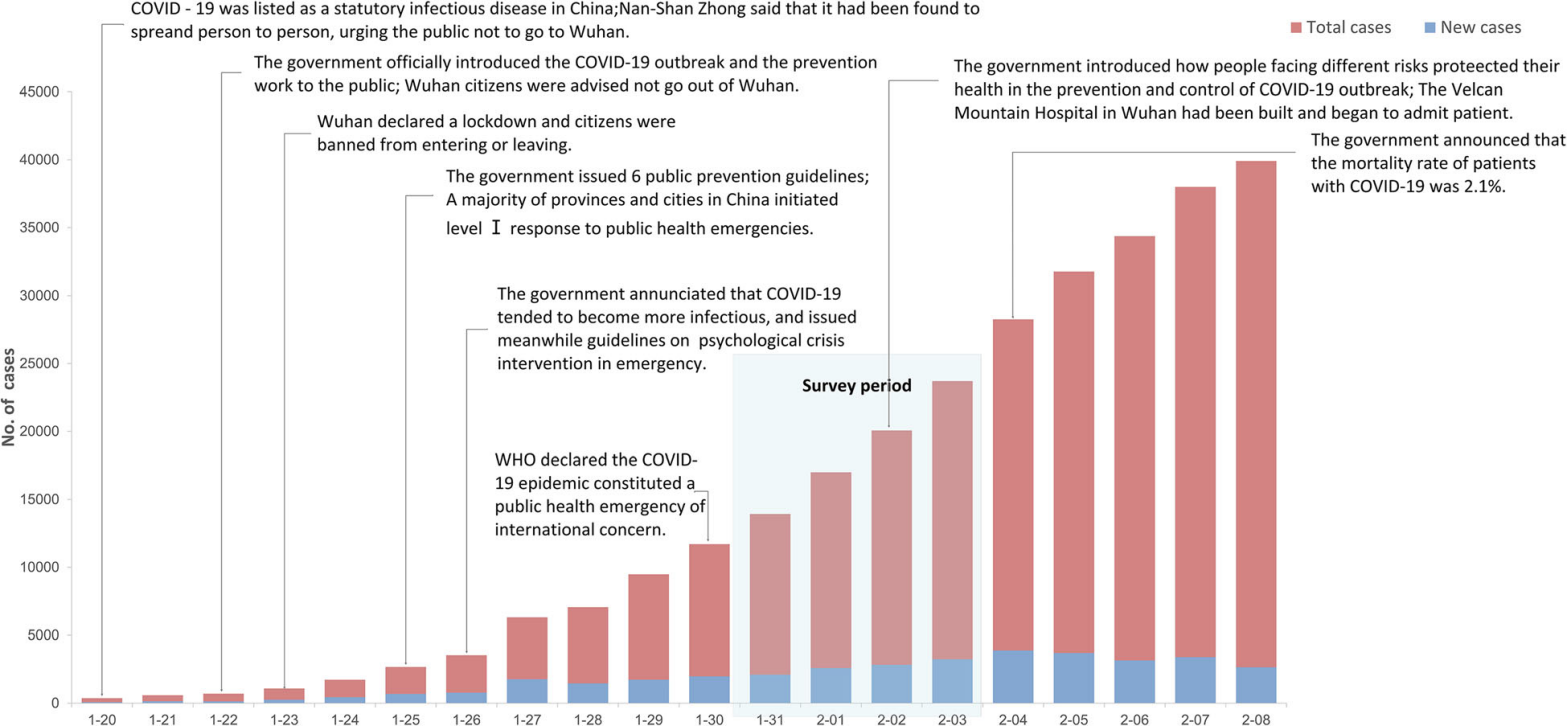
COVID-19 epidemic curve and dates of key events in China. On January 24, the Chinese Spring Festival began. The public health emergency I response was initiated in most areas of China on January 25, and the government began to intervene in people’s lives and travel on a large scale in an attempt to prevent the epidemic from spreading further. However, the number of confirmed cases continued to rise, and it was not until February 6 that the number of new cases began to decline. The above data were sourced from China CDC and media or official reports

The survey period was from January 30, 2020 to February 3, 2020. Figure 1 shows the COVID-19 epidemic curve in China and dates of key events. In total, 620 questionnaires were retrieved; 12 questionnaires were excluded because of a previously diagnosed psychological illness. Among the 608 valid questionnaires included in our study, 153 respondents did not complete the SCL-90, and only 455 respondents completed all survey scales. Respondents’ demographic characteristics are shown in Table 1. Respondents were from 28 provinces and cities around China and were distributed across different ages, occupations, and education levels; therefore, we believe that these respondents could represent the Chinese public. We found that during the peak of the COVID-19 epidemic, respondents’ state anxiety scores, trait anxiety scores, SDS index scores, and SCL-90 total scores did not exceed the normal range according to the healthy norm results for these scales (Supplementary Table 1). The analysis of variance by age group, gender, and scale scores showed there were significant differences in STAI scores across age groups (Supplementary Tables 2.1 and 2.2), which was consistent with the STAI normal model. The SDS index scores also varied across age groups. However, the SCL-90 scores did not show any differences by age group or gender.

**Table 1:** The demographic characteristics of the respondents

Variable		Number (Total, *n* = 608)	Proportion (%)
Gender	Male	251	41.3
	Female	357	58.7
Age group (years)	Below 18 (including 18)	34	5.6
	19–39	321	52.8
	40–49	149	24.5
	50–69	99	16.3
	Above 70 (including 70)	5	0.8
Occupation	Government, enterprises and institutions	136	22.4
	Worker	123	20.2
	Student	118	19.4
	Businessman	58	9.5
	Medical staff	58	9.5
	Unemployed	27	4.4
	Other	88	14.5
Education level	Primary school degree	5	0.8
	Junior high school degree	54	8.9
	High school degree	80	13.2
	College degree	389	64.0
	Master or doctor degree	80	13.2
City or region	Hubei province	61	10.0
	Guangdong province	264	43.4
	Other province or regions	283	46.5
History of epidemiological exposure^a^	Yes	59	9.7
	No	549	90.3
Someone around you diagnosed as (suspected) epidemiological carrier	Yes	19	3.1
	No	589	96.9
Someone around you who is a medical staff withstanding SARI^b^	Yes	281	46.2
	No	227	53.8

^a^ History of epidemiological exposure means he/she has ever been to any epidemic area in Hubei, or been exposed to people there

^b^*SARI* severe acute respiratory infection

### Public psychological status

The healthy norm results of the three scales were used as the criteria to assess psychological status. The age range of STAI norm results was 19–69 years; therefore, we excluded 39 questionnaires for respondents aged < 18 or > 70 years. According to the healthy norm results of the SDS and SCL-90, depression was classified by an SDS index score ≥ 50, and the psychological abnormality was classified by a SCL-90 total score ≥ 160. Respondents’ psychological status (state anxiety, trait anxiety, depression, and psychological abnormalities) is shown in Table 2.

**Table 2:** The proportion of respondents with anxiety, depression and psychological abnormalities

Variable		*n*	Proportion (%)
State anxiety (*n* = 569)^a^	Yes	90	15.8
	No	479	84.2
Trait anxiety (*n* = 569)^a^	Yes	23	4.0
	No	546	96.0
Depression (*n* = 608)	Yes	165	27.1
	No	443	72.9
Psychology abnormal (*n* = 455)^b^	Yes	35	7.7
	No	420	92.3

^a^ The age range of ST-AI norm result was from 19 to 69, 39 questionnaires under 18 or above 70 years of age were excluded, 569 cases were enrolled in the analysis

^b^ Among 608 valid questionnaires, 153 didn’t complete SCL-90

### Proportion of respondents by psychological status

More respondents had state anxiety (15.8%, 90/569) than trait anxiety (4.0%, 23/569) (*P* < 0.001; Table 3). The average score for state anxiety was also higher than that for trait anxiety (Supplementary Table 3), which remained consistent. We also found a high proportion of respondents with depression (27.1%), and 7.7% respondents had psychological abnormalities.

**Table 3:** The chi-square result between State-anxiety and Trait-anxiety

Variable		Trait anxiety, *n* (%)		Total	*χ*^2^	*P* value
		Yes	No	*n* (%)		
State anxiety	Yes	21 (91.3)	69 (12.6)	90 (15.8)	96.752	< 0.001
	No	2 (8.7)	477 (87.4)	479 (84.2)		
	Total	23	546	569		

### Anxiety by age groups

Both state and trait anxiety were more common in females than in males. Respondents’ psychological status also differed across age groups. Respondents aged 19–39 years appeared to be more prone to state anxiety (43.3%), depression (61.8%), and psychological abnormalities (74.3%) than other respondents. Those aged 40–49 years had the lowest rates of state anxiety (15.6%) and trait anxiety (4.4%). The proportion of trait anxiety among respondents aged 50–69 years was 73.9%.

### Influence of other factors on psychological status

The proportions of respondents with trait anxiety and depression differed by their occupation. Differences in education level and region were only found in trait anxiety. People with a history of epidemiology (including those who had visited Hubei province or come into contact with people from epidemic areas) were less likely to be anxious than those who had not. Neither the presence of a confirmed/suspected epidemiological carrier nor the presence of a medical worker around respondents increased levels of anxiety or psychological abnormalities. Moreover, the impact of the COVID-19 outbreak on respondents’ work or life had no effect on their psychological status. However, those who were more worried about being infected with COVID-19 had a higher proportion of state anxiety. Of the respondents with state anxiety, 33.3% were “very worried” about being infected with COVID-19 and only 2.2% were “not worried at all.” Those that were more confident about overcoming the epidemic outbreak appeared to have lower rates of state anxiety and depression compared with other respondents (Table 4).

**Table 4:** Psychological status of the public under the epidemic of COVID-19 in China

Variable		State anxiety				Trait anxiety				Depression				Psychology abnormality ^b^			
		Total (*n* = 569) *n* (%)	*N*o (*n* = 479) *n* (%)	Yes (*n* = 90) *n* (%)	*P* value	Total (*n* = 569) *n* (%)	*N*o (*n* = 546) *n* (%)	Yes (*n* = 23) *n* (%)	*P* value	Total (*n* = 608) *n* (%)	*N*o (*n* = 443) *n* (%)	Yes (*n* = 165) *n* (%)	*P* value	Total (*n* = 455) *n* (%)	*N*o (*n* = 420) *n* (%)	Yes (*n* = 35) *n* (%)	*P* value
Gender	Male	240 (42.2)	211 (44.1)	29 (32.2)	0.037	240 (42.2)	235 (43.0)	5 (21.7)	0.043	251 (41.3)	183 (41.3)	68 (41.2)	0.983	182 (40.0)	171 (40.7)	11 (31.4)	0.281
	Female	329 (57.8)	268 (56.0)	61 (67.8)		329 (57.8)	311 (57.0)	18 (78.3)		357 (58.7)	260 (58.7)	97 (58.8)		273 (60.0)	249 (59.3)	24 (68.6)	
Age group (years)^a^	Below 18 (including 18)	_	_	_	0	_	_	_	0	34 (5.6)	21 (4.7)	13 (7.9)	0.006	24 (5.3)	21 (5.0)	3 (8.6)	0.042
	19–39	321 (56.4)	282 (58.9)	39 (43.3)		321 (56.4)	316 (57.9)	5 (21.7)		321 (52.8)	219 (49.4)	102 (61.8)		239 (52.5)	213 (50.7)	26 (74.3)	
	40–49	149 (26.2)	135 (28.2)	14 (15.6)		149 (26.2)	148 (27.1)	1 (4.4)		149 (24.5)	122 (27.5)	27 (16.4)		117 (25.7)	113 (26.9)	4 (11.4)	
	50–69	99 (17.4)	62 (12.9)	37 (41.1)		99 (17.4)	82 (15.0)	17 (73.9)		99 (16.3)	76 (17.2)	23 (13.9)		71 (15.6)	69 (16.4)	2 (5.7)	
	Above 70 (including 70)	_	_	_		_	_	_		5 (0.8)	5 (1.1)	0 (0.0)		4 (0.9)	4 (1.0)	0 (0.0)	
Occupation	Government, enterprises and institutions	136 (23.9)	114 (23.8)	22 (24.4)	0.23	136 (23.9)	134 (24.5)	2 (8.7)	0.002	136 (22.4)	111 (25.1)	25 (15.2)	0	99 (21.8)	96 (22.9)	3 (8.6)	0.181
	Worker	122 (21.4)	109 (22.8)	13 (14.4)		122 (21.4)	117 (21.4)	5 (21.7)		123 (20.2)	90 (20.3)	33 (20.0)		96 (21.1)	89 (21.2)	7 (20.0)	
	Student	84 (14.8)	73 (15.2)	11 (12.2)		84 (14.8)	83 (15.2)	1 (4.4)		118 (19.4)	67 (15.1)	51 (30.9)		76 (16.7)	67 (16.0)	9 (25.7)	
	Businessman	58 (10.2)	49 (10.2)	9 (10.0)		58 (10.2)	56 (10.3)	2 (8.7)		58 (9.5)	44 (9.9)	14 (8.5)		50 (11.0)	45 (10.7)	5 (14.3)	
	Medical staff	58 (10.2)	48 (10.0)	10 (11.1)		58 (10.2)	56 (10.3)	2 (8.7)		58 (9.5)	42 (9.5)	16 (9.7)		46 (10.1)	42 (10.0)	4 (11.4)	
	Unemployed	27 (4.8)	19 (4.0)	8 (8.9)		27 (4.8)	22 (4.0)	5 (21.7)		27 (4.4)	17 (3.8)	10 (6.1)		24 (5.3)	20 (4.8)	4 (11.4)	
	Other	84 (14.8)	67 (14.0)	17 (18.9)		84 (14.8)	78 (14.3)	6 (26.1)		88 (14.5)	72 (16.3)	16 (9.7)		64 (14.1)	61 (14.5)	3 (8.6)	
Education level	Primary school degree	2 (0.4)	2 (0.4)	0 (0.0)	0.777	2 (0.4)	2 (0.4)	0 (0.0)	0.027	5 (0.8)	5 (1.1)	0 (0.0)	0.173	4 (0.9)	4 (1.0)	0 (0.0)	0.95
	Junior high school degree	37 (6.5)	30 (6.3)	7 (7.8)		37 (6.5)	34 (6.2)	3 (13.0)		54 (8.9)	33 (7.5)	21 (12.7)		43 (9.5)	40 (9.5)	3 (8.6)	
	High school degree	74 (13.0)	60 (12.5)	14 (15.6)		74 (13.0)	67 (12.3)	7 (30.4)		80 (13.2)	61 (13.8)	19 (11.5)		68 (15.0)	62 (14.8)	6 (17.1)	
	College degree	377 (66.3)	318 (66.4)	59 (65.6)		377 (66.3)	364 (66.7)	13 (56.5)		389 (64.0)	287 (64.8)	102 (61.8)		290 (63.7)	267 (63.6)	23 (65.7)	
	Master or doctor degree	79 (13.9)	69 (14.4)	10 (11.1)		79 (13.9)	79 (14.5)	0 (0.0)		80 (13.2)	57 (12.9)	23 (13.9)		50 (11.0)	47 (11.2)	3 (8.6)	
City or region	Hubei province	49 (8.6)	38 (7.9)	11 (12.2)	0.134	49 (8.6)	43 (7.9)	6 (26.1)	0.006	61 (10.0)	41 (9.3)	20 (12.1)	0.298	40 (8.8)	39 (9.3)	1 (2.9)	0.178
	Guangdong province	245 (43.1)	214 (44.7)	31 (34.4)		245 (43.1)	235 (43.0)	10 (43.5)		264 (43.4)	200 (45.2)	64 (38.8)		203 (44.6)	190 (45.2)	13 (37.1)	
	Other province or regions	275 (48.3)	227 (47.4)	48 (53.3)		275 (48.3)	268 (49.1)	7 (30.4)		283 (46.6)	202 (45.6)	81 (49.1)		212 (46.6)	191 (45.5)	21 (60.0)	
History of epidemiological exposure	Yes	54 (9.5)	38 (7.9)	16 (17.8)	0.003	54 (9.5)	46 (8.4)	8 (34.8)	0	59 (9.7)	38 (8.6)	21 (12.7)	0.124	40 (8.8)	40 (9.5)	0 (0.0)	0.109
	No	515 (90.5)	441 (92.1)	74 (82.2)		515 (90.5)	500 (91.6)	15 (65.2)		549 (90.3)	405 (91.4)	144 (87.3)		415 (91.2)	380 (90.5)	35 (100.0)	
Someone around you diagnosed as (suspected) epidemiological carrier	Yes	17 (3.0)	12 (2.5)	5 (5.6)	0.222	17 (3.0)	15 (2.8)	2 (8.7)	0.147	19 (3.1)	10 (2.3)	9 (5.5)	0.044	12 (2.6)	11 (2.6)	1 (2.9)	1
	No	552 (97.0)	467 (97.5)	85 (94.4)		552 (97.0)	531 (97.3)	21 (91.3)		589 (96.9)	433 (97.7)	156 (94.6)		443 (97.4)	409 (97.4)	34 (97.1)	
Someone around you who is a medical staff withstanding SARI	Yes	271 (47.6)	225 (47.0)	46 (51.1)	0.471	271 (47.6)	261 (47.8)	10 (43.5)	0.684	281 (46.2)	208 (47.0)	73 (44.2)	0.551	219 (48.1)	202 (48.1)	17 (48.6)	0.957
	No	298 (52.4)	254 (53.0)	44 (48.9)		298 (52.4)	285 (52.2)	13 (56.5)		327 (53.8)	235 (53.1)	92 (55.8)		236 (51.9)	218 (51.9)	18 (51.4)	
Knowledge about COVID-19	Nothing	12 (2.1)	7 (1.5)	5 (5.6)	0.004	12 (2.1)	10 (1.8)	2 (8.7)	0.021	12 (2.0)	7 (1.6)	5 (3.0)	0.028	11 (2.4)	10 (2.4)	1 (2.9)	0.184
	A little	25 (4.4)	21 (4.4)	4 (4.4)		25 (4.4)	22 (4.0)	3 (13.0)		29 (4.8)	15 (3.4)	14 (8.5)		20 (4.4)	16 (3.8)	4 (11.4)	
	Some	130 (22.9)	101 (21.1)	29 (32.2)		130 (22.9)	123 (22.5)	7 (30.4)		140 (23.0)	97 (21.9)	43 (26.1)		96 (21.1)	87 (20.7)	9 (25.7)	
	Much	300 (52.7)	256 (53.4)	44 (48.9)		300 (52.7)	291 (53.3)	9 (39.1)		317 (52.1)	239 (54.0)	78 (47.3)		236 (51.9)	219 (52.1)	17 (48.6)	
	Very much	102 (17.9)	94 (19.6)	8 (8.9)		102 (17.9)	100 (18.3)	2 (8.7)		110 (18.1)	85 (19.2)	25 (15.2)		92 (20.2)	88 (21.0)	4 (11.4)	
The impact of the COVID-19 outbreak on your life or work	Almost nothing	16 (2.8)	14 (2.9)	2 (2.2)	0.329	16 (2.8)	15 (2.8)	1 (4.4)	0.83	23 (3.8)	18 (4.1)	5 (3.0)	0.387	18 (4.0)	17 (4.1)	1 (2.9)	0.09
	Some	154 (27.1)	136 (28.4)	18 (20.0)		154 (27.1)	148 (27.1)	6 (26.1)		164 (27.0)	123 (27.8)	41 (24.9)		121 (26.6)	114 (27.1)	7 (20.0)	
	Much	308 (54.1)	256 (53.4)	52 (57.8)		308 (54.1)	297 (54.4)	11 (47.8)		327 (53.8)	240 (54.2)	87 (52.7)		242 (53.2)	226 (53.8)	16 (45.7)	
	Very much	91 (16.0)	73 (15.2)	18 (20.0)		91 (16.0)	86 (15.8)	5 (21.7)		94 (15.5)	62 (14.0)	32 (19.4)		74 (16.3)	63 (15.0)	11 (31.4)	
Degree of worry about epidemiological infection	Not at all	46 (8.1)	44 (9.2)	2 (2.2)	0	46 (8.1)	44 (8.1)	2 (8.7)	0.308	50 (8.2)	48 (8.3)	2 (6.9)	0.175	34 (7.5)	32 (7.6)	2 (5.7)	0.171
	A little	310 (54.5)	273 (57.0)	37 (41.1)		310 (54.5)	299 (54.8)	11 (47.8)		336 (55.3)	324 (56.0)	12 (41.4)		250 (55.0)	236 (56.2)	14 (40.0)	
	Much	118 (20.7)	97 (20.3)	21 (23.3)		118 (20.7)	115 (21.1)	3 (13.0)		121 (19.9)	115 (19.9)	6 (20.7)		92 (20.2)	83 (19.8)	9 (25.7)	
	Very much	95 (16.7)	65 (13.6)	30 (33.3)		95 (16.7)	88 (16.1)	7 (30.4)		101 (16.6)	92 (15.9)	9 (31.0)		79 (17.4)	69 (16.4)	10 (28.6)	
Confidence about overcoming this outbreak	Nothing	5 (0.9)	4 (0.8)	1 (1.1)	0	5 (0.9)	5 (0.9)	0 (0.0)	0.186	5 (0.8)	3 (0.7)	2 (1.2)	0	3 (0.7)	3 (0.7)	0 (0.0)	0.077
	A little	131 (23.0)	91 (19.0)	40 (44.4)		131 (23.0)	123 (22.5)	8 (34.8)		139 (22.9)	83 (18.7)	56 (33.9)		99 (21.8)	88 (21.0)	11 (31.4)	
	Much	164 (28.8)	138 (28.8)	26 (28.9)		164 (28.8)	155 (28.4)	9 (39.1)		175 (28.8)	123 (27.8)	52 (31.5)		130 (28.6)	116 (27.6)	14 (40.0)	
	Very much	269 (47.3)	246 (51.4)	23 (25.6)		269 (47.3)	263 (48.2)	6 (26.1)		289 (47.5)	234 (52.8)	55 (33.3)		223 (49.0)	213 (50.7)	10 (28.6)	

^a^The age range of ST-AI norm result was from 19 to 69, 39 questionnaires under 18 or above 70 years of age were excluded, 569 cases were enrolled in the analysis

^b^ Among 608 valid questionnaires, 153 didn’t complete SCL-90

### Behavior changes

During the COVID-19 epidemic, most (93.3%) respondents avoided going to public places (behavior change 1) (Table 5). Even during the Spring Festival, which is the most important traditional festival in China, almost all respondents reduced festival-related activities (behavior change 2) to avoid contact with others. In addition, at least 70.9% of respondents chose to take three or more preventive measures to avoid infection. The three most commonly used prevention measures were “making fewer trips outside and avoiding contact (98.0%),” “wearing a mask (83.7%),” and “hand hygiene (82.4%)” (Supplement Figure 1). Surprisingly, respondents’ anxiety did not appear to be related to public behavior change and preventive measures. Fewer respondents with depression took preventive measures compared with those without depression. In addition, those with psychological abnormalities appeared to be less likely to avoid spring festival-related activities and preventive measures compared with other respondents.

**Table 5:** Chi-square analysis results between behavior changes and different psychological status under the epidemic of COVID-19 in China

Variable		State anxiety, *n* (%)^d^				Trait anxiety, *n* (%)^d^				Depression, *n* (%)				Psychology abnormal, *n* (%)^e^			
		Total (*n* = 569)	No (*n* = 479)	Yes (*n* = 90)	*P* value	Total (*n* = 569)	No (*n* = 546)	Yes (*n* = 23)	*P* value	Total (*n* = 608)	No (*n* = 443)	Yes (*n* = 165)	*P* value	Total (*n* = 455)	No (*n* = 420)	Yes (*n* = 35)	*P* value
Behavior change 1^a^	Never	531 (93.3)	444 (92.7)	87 (96.7)	0.166	531 (93.3)	510 (93.4)	21 (91.3)	0.975	567 (93.3)	415 (93.7)	152 (92.1)	0.496	430 (94.5)	398 (94.8)	32 (91.4)	0.656
	Sometimes	38 (6.7)	35 (7.3)	3 (3.3)		38 (6.7)	36 (6.6)	2 (8.7)		41 (6.7)	28 (6.3)	13 (7.9)		25(5.5)	22 (5.2)	3 (8.6)	
	Same as usual	0 (0.0)	0 (0.0)	0 (0.0)		0 (0.0)	0 (0.0)	0 (0.0)		0 (0.0)	0 (0.0)	0 (0.0)		0 (0.0)	0 (0.0)	0 (0.0)	
	More than usual	0 (0.0)	0 (0.0)	0 (0.0)		0 (0.0)	0 (0.0)	0 (0.0)		0 (0.0)	0 (0.0)	0 (0.0)		0 (0.0)	0 (0.0)	0 (0.0)	
Behavior change 2^b^	Never	510 (89.6)	430 (89.8)	80 (88.9)	0.868	510 (89.6)	488 (89.4)	22 (95.7)	0.624	546 (89.8)	402 (90.7)	144 (87.3)	0.223	416 (91.4)	389 (92.6)	27 (77.1)	0.005
	Sometimes when necessary	58 (10.2)	48 (10.0)	10 (11.1)		58 (10.2)	57 (10.4)	1 (4.4)		60 (9.9)	40 (9.0)	20 (12.1)		38 (8.4)	30 (7.1)	8 (22.9)	
	Same as Usual	0 (0.0)	0 (0.0)	0 (0.0)		0 (0.0)	0 (0.0)	0 (0.0)		1 (0.2)	1 (0.2)	0 (0.0)		0 (0.0)	0 (0.0)	0 (0.0)	
	More than usual	1 (0.2)	1 (0.2)	0 (0.0)		1 (0.2)	1 (0.2)	0 (0.0)		1 (0.2)	0 (0.0)	1 (0.6)		1 (0.2)	1 (0.2)	0 (0.0)	
Preventive measures ^c^	1	85 (14.9)	71 (14.8)	14 (15.6)	0.78	85 (14.9)	80 (14.7)	5 (21.7)	0.787	88 (14.5)	50 (11.3)	38 (23.0)	0.004	59 (13.0)	51 (12.1)	8 (22.9)	0.02
	2	31 (5.5)	24 (5.0)	7 (7.8)		31 (5.5)	30 (5.5)	1 (4.4)		38 (6.3)	26 (5.9)	12 (7.3)		24 (5.3)	19 (4.5)	5 (14.3)	
	3	403 (70.8)	340 (71.0)	63 (70.0)		403 (70.8)	389 (71.3)	14 (60.9)		431 (70.9)	330 (74.5)	101 (61.2)		333 (73.2)	315 (75.0)	18 (51.4)	
	4	49 (8.6)	43 (9.0)	6 (6.7)		49 (8.6)	46 (8.4)	3 (13.0)		50 (8.2)	36 (8.1)	14 (8.5)		38 (8.4)	34 (8.1)	4 (11.4)	
	5	1 (0.2)	1 (0.2)	0 (0.0)		1 (0.2)	1 (0.2)	0 (0.0)		1 (0.2)	1 (0.2)	0 (0.0)		1 (0.2)	1 (0.2)	0 (0.0)	

^a^ Behavior change 1: go out to public place

^b^ Behavior change 2: attend Spring Festival-relative activities

^c^ Preventive measures: making fewer trips outside and avoiding contact, hand hygiene, wearing a mask, taking traditional Chinese medicine and others

^d^ The age ranges of ST-AI norm results4 only includes 19–69, so we exclude 39 whose age under 18 and above 70

^e^ Among 608 valid questionnaires, 53 didn’t complete SCL-90

### Other psychological abnormalities: phobia

The SCL-90 covers 10 different psychological abnormality factors. According to the results for the normal model of the scale, we defined a score of ≥ 2 for each factor as corresponding to abnormal symptoms. The results are shown in Table 6. The scores for the phobia factor were the highest, with an average score of 1.29 ± 0.47, which also exceeded Chinese healthy norm results (1.23 ± 0.41) [28]. About 10.1% of respondents suffered from phobia (Supplementary Table 4), which indicated that a phobia state may be present in the wider public.

**Table 6:** The results of 455 respondents’ SCL-90 factors scores compared with norm result of Chinese healthy (mean ± std. deviation)

Variable	Chinese healthy	Respondents in this study
Somatization factor	1.37 ± 0.48	1.18 ± 0.35
Obsession factor	1.62 ± 0.58	1.35 ± 0.47
Interpersonal sensitivity factor	1.65 ± 0.51	1.27 ± 0.45
Depression factor	1.50 ± 0.59	1.24 ± 0.45
Anxiety factor	1.39 ± 0.43	1.21 ± 0.40
Hostile factor	1.48 ± 0.56	1.19 ± 0.40
Phobia factor	1.23 ± 0.41	1.29 ± 0.47
Paranoid factor	1.43 ± 0.57	1.18 ± 0.39
Psychotic factor	1.29 ± 0.42	1.18 ± 0.39

## Discussion

Most previous studies in this area focused on the psychological status of patients [29] or medical staff [30–32] during the epidemic, and little attention has been directed to the psychological status and behavior changes of the general public. Our survey found that the ratio of overall state anxiety among respondents was 15.8%, which was greater than that of trait anxiety, suggesting that the epidemic had caused some anxiety. Previous studies reported the public experienced varying levels of anxiety during previous pandemics [33–38]. In our study, women appeared to be more prone to anxiety than men, which may be related to their sensitivity to psychological stress. We found the psychological status of different age groups showed different tendencies during the epidemic. It appeared that young people were more likely to suffer from state anxiety, depression, and psychological abnormalities when faced with the epidemic. The reasons for this are complex. This segment of the population tends to obtain more information about such issues from the media. They also have the main responsibility for social productivity and their family, and therefore bear more psychological pressure. Previous studies found that young people had higher anxiety, depression, and stress scores than older people [39]. We also found that older adults had a higher proportion of trait anxiety than other age groups, accounting for 73.9% of the total trait anxiety population. Specific reasons for this result need further research. However, the government needs to make different decisions based on the gender and age characteristics of the population when formulating psychological interventions. There were no significant differences in state anxiety, depression, or psychological abnormalities among people with different education levels, possibly because the public had uniform susceptibility to the epidemic. Higher literacy does not appear to help an individual deal with psychological stress better than lower literacy. The proportion of population with anxiety, depression, and psychological abnormalities in Hubei, the worst-hit province, did not significantly differ from Guangdong or other provinces. This may be explained by the small number of respondents from Hubei province. Because of the outbreak, we were unable to find a suitable partner in Hubei province (especially in Wuhan) to help us complete the online questionnaire. The publicity from the government and media meant that 23.0% of respondents reported they had “some” knowledge of COVID-19, 52.1% had “much” knowledge, and 18.1% had “very much” knowledge. This suggested that the general public had a sufficient understanding of COVID-19. It is important to note that people who knew more about COVID-19 were less likely to experience anxiety, depression, and psychological abnormalities than those with low knowledge. This may be one reason for the low overall anxiety score of the study population. Public understanding of the epidemic is an important consideration for psychological interventions. In addition, the level of public trust in the government and medical institutions, and the level of public anxiety have a significant negative impact [40]. Scientists need to keep working to determine the pathogenesis, treatment, and vaccine development for COVID-19. In addition, the government needs to honestly and correctly report the real epidemic situation to reduce public anxiety, fear, and other negative psychological states; this may help in gaining public trust.

It seems natural that people are more prone to anxiety, depression, and fear when facing unknown things or diseases. The more worried people are, the more anxious they become. Anxiety is the fear of expected danger, and panic is the spread of anxiety among a group. In this context, individual anxiety constantly spread through the rapid transmission of information, and evolved into group anxiety and panic. As the number of confirmed cases and deaths from COVID-19 increase, the public’s psychological state is likely to worsen. However, moderate anxiety could increase awareness of disease prevention and reduce the incidence of disease. A study from Hong Kong noted that a certain level of anxiety could prompt people to take more preventive measures to reduce the speed of SARS transmission [34]. Therefore, some degree of anxiety may not be “bad”. However, addressing moderate anxiety remains difficult. A previous study showed that the H1N1 epidemic threatened the public’s physical health, but also caused psychological distress; these results differed based on a series of assessment and coping factors [41].

On January 25, 2020, after most provinces and cities in China successively initiated the level I response to the public health emergency, the government began to intervene in public lives and travel on a large scale. This intervention came with certain “mandatory” requirements. The government required the public to follow specific behaviors. If you do not perform these behaviors, it will be considered a violation of the law. As a result, the majority of respondents followed specific behaviors required by the authorities; 93.3% of our respondents said they “never” went to public places, and 89.6% “never” attended Spring Festival-related activities. Therefore, in our investigation, behavior changes and preventive measures adopted by the public were not related to their psychological status. Regardless of whether it is advisable to restrict individual freedom, such restrictions are beneficial to control further expansion of the epidemic. Previous evidence has shown that encouraging the public to take specific health-related actions is useful to curb epidemics [42–45]. When an epidemic is under control, it is likely that the psychological status of the general public will naturally return to pre-epidemic status.

In our study, the sample was not adjusted to reflect the proportion of the population in terms of gender, age, and region. This was because it was important to evaluate public psychological stress in a timely manner. In addition, there were insufficient respondents from Wuhan and other cities in Hubei province. Therefore, we need to be careful in interpreting our results. A second limitation was that we used an online questionnaire survey to reflect the strict measures around social distancing; however, there is no guarantee that the questionnaire responses were not distorted. The third limitation was that the cross-sectional nature of the study means it cannot reflect trends of psychological changes of people in China. Finally, our study found that respondents’ state anxiety scores, trait anxiety scores, SDS index scores, and SCL-90 total scores did not exceed the normal range. However, this could be attributable to the lack of sensitivity of the instruments used in this study.

## Conclusions

In China, public behavior changes and prevention measures are greatly affected by the strong intervention of the Chinese government. The majority of people follow specific behaviors required by the authorities, but it will take time to observe the effects of these behaviors on the epidemic. However, some Chinese people are experiencing anxiety, depression, and other psychological abnormalities during this epidemic. The government needs to pay more attention to the psychological status of the public, especially those aged 19–39 years. This age group appears likely to experience psychological stress when faced with an infectious disease epidemic. Based on the public psychological status during the COVID-19 epidemic, we suggest that policymakers consider making appropriate adjustments to reflect gender and age characteristics when formulating psychological intervention measures. In addition, the government should share as much information as possible with the public, such as knowledge about COVID-19, daily outbreak status, and the government’s epidemic prevention strategy; this may help to relieve psychological stress. We hope that this preliminary survey can provide some guidance for psychological interventions for the Chinese population.

## Supplementary information

**Additional file 1.**

## Data Availability

Not applicable.

## Abbreviations

SARS: Severe acute respiratory syndrome
COVID-19: Coronavirus disease 2019
STAI: State-Trait Anxiety Inventory
SDS: Self-rating Depression Scale
SCL-90: Symptom Checklist-90
