# Supplementary material for: Psychological status and behavior changes of the public during the COVID-19 epidemic in China
Source: Infect Dis Poverty. 2020 May 29;9:58. doi: 10.1186/s40249-020-00678-3 (PMC7256340; doi:10.1186/s40249-020-00678-3)
Supplement: Supplementary file 1 — Additional file 1. [file 40249_2020_678_MOESM1_ESM.zip › Supplementary Material 0510.docx]

Supplementary Material

# Supplementary Figures and Tables

| **Supplementary Table 1** The results of the ST-AI scores, SDS index scores and SCL-90 total scores of the respondents | | |
| --- | --- | --- |
| Variable | Mean±Std. Deviation | Mean 95% CI(LL-UL) |
| S-AI scores (n=608) | 42.91±8.96 | 42.20-43.68 |
| T-AI scores (n=608) | 40.75±8.15 | 40.10-41.40 |
| SDS index scores(n=608) | 43.00±11.72 | 33.55-35.04 |
| SCL-90 total scores(n=455)^a^ | 110.95±33.18 | 107.9-114.00 |
| a. Among 608 valid questionnaires, 153 didn’t complete SCL-90. | | |

| **Supplementary Table 2.1** ST-AI scores and SDS index scores of different age groups and genders variance analysis results of 608 respondents | | | | | | | | | | |
| --- | --- | --- | --- | --- | --- | --- | --- | --- | --- | --- |
|  | | S-AI scores | |  | T-AI scores | |  | SDS index scores | |  |
|  | Gender | male | female |  | male | female |  | male | female |  |
| Age group | Below 18 ( including 18) (n=34) | 40.38±4.72 | 44.23±7.88 | *P*=0.010 | 38.88±4.32 | 43.38±7.27 | *P*=0.000 | 35.25±5.80 | 50.42±12.93 | *P*=0.000 |
|  | 19~39(n=321) | 43.34±8.68 | 44.55±9.50 |  | 43.02±7.56 | 42.42±7.71 |  | 46.37±12.82 | 44.27±11.47 |  |
|  | 40~49(n=149) | 40.54±9.27 | 42.17±8.04 |  | 37.27±8.13 | 39.72±6.71 |  | 37.76±10.20 | 41.16±10.42 |  |
|  | 50~69(n=99) | 41.36±8.36 | 41.81±8.78 |  | 38.22±8.92 | 37.54±9.04 |  | 41.93±10.10 | 40.02±10.31 |  |
|  | Above 70 (including 70) (n=5) | 37.00±13.11 | 36.50±2.12 |  | 34.67±14.57 | 28.00±11.31 |  | 32.00±6.56 | 28.00±4.24 |  |
|  |  | *P*=0.080 | |  | *P*=0.610 | |  | *P*=0.948 | |  |

|  | **Supplementary Table 2.2** SCL-90 total scores of different age groups and genders variance analysis results of 455 respondents | | | |
| --- | --- | --- | --- | --- |
|  | Gender | SCL-90 total scores | |  |
|  |  | male | female |  |
| Age group | Below 18 (including 18) (n=24) | 101.14±16.07 | 130.24±36.28 | *P*=0.070 |
|  | 19~39(n=239) | 113.93±43.78 | 114.32±33.72 |  |
|  | 40~49(n=117) | 102.05±17.13 | 110.28±36.55 |  |
|  | 50~69(n=71) | 100.40±8.83 | 108.05±27.39 |  |
|  | Above 70 (including 70) (n=4) | 102.00±10.44 | 103.00±null |  |
|  |  | *P*=0.110 | |  |

| **Supplementary Table 3** S-AI and T-AI independent t test analysis results | | | |
| --- | --- | --- | --- |
| group | (Mean±Std. Deviation) | t | *P* value |
| S-AI scores(n=608) | 42.91±8.96 | 4.416 | 0.000 |
| T-AI scores(n=608) | 40.75±8.15 |  |  |

| **Supplementary Table 4** SCL-90 factors abnormal in the respondents of 455 respondents | | | |
| --- | --- | --- | --- |
| Variable | | N | Percent(%) |
| Psychology abnormal | yes | 35 | 7.7 |
|  | no | 420 | 92.3 |
| Somatization factor abnormal | yes | 17 | 3.7 |
|  | no | 438 | 96.3 |
| Obsession factor abnormal | yes | 39 | 8.6 |
|  | no | 416 | 91.4 |
| Interpersonal sensitivity factor abnormal | yes | 33 | 7.3 |
|  | no | 422 | 92.8 |
| Depression factor abnormal | yes | 29 | 6.4 |
|  | no | 426 | 93.6 |
| Anxiety factor abnormal | yes | 22 | 4.8 |
|  | no | 433 | 95.2 |
| Hostile factor abnormal | yes | 27 | 5.9 |
|  | no | 428 | 94.1 |
| Phobia factor abnormal | yes | 46 | 10.1 |
|  | no | 409 | 89.9 |
| Paranoid factor abnormal | yes | 24 | 5.3 |
|  | no | 431 | 94.7 |
| Psychotic factor abnormal | yes | 22 | 4.8 |
|  | no | 433 | 95.2 |
|  | total | 455 | 100.0 |

## Supplementary Figures

**Supplementary Figure 1** The preventive measures of respondents against for COVID-19.

Among the 608 valid questionnaires, 98.0% of the respondents chose to reduce going out and avoiding contact to prevent infection, 83.7% would wear masks, and 82.4% would perform hand hygiene. No one chose not to take preventive measures.

**Investigation of the psychological status of the public under the outbreak** **of COVID-19**

The outbreak of COVID-19 emerged before the Spring Festival, which has caused serious damage to our country. The Fifth Affiliated Hospital of Sun Yat-sen University infection prevention and control center in order to figure out the psychological status of the public under the outbreak, issuing this questionnaire to evaluate the psychological status in the face of the epidemic. To protect everyone's physical and mental health, please complete these questions seriously. This questionnaire will take about **10-20** **minutes** to finalize.

1. Your Gender:

| ○Male | ○Female |  |  |  |  |  |  |
| --- | --- | --- | --- | --- | --- | --- | --- |

1. Your age group:

○Under 18 (including 18 years old)

○19 to 39

○40 to 49

○ 65 to 69

○Over 70 (including 70 years old)

3. Your current occupation:

| ○Student |
| --- |
| ○Worker |
| ○Self-employed, business personnel |
| ○Government, enterprise and institutions personnel |
| ○Medical staff |
| ○Unemployed personnel |
| ○Other |

4. What city and region you are currently in: _________________________________

(fill in the blanks)

5. Your level of education:

| ○Primary school degree |
| --- |
| ○Junior high school degree |
| ○High school degree |
| ○College degree |
| ○Master or doctor degree |

6. Have you ever had mental health problems such as mental illness, such as anxiety and depression, etc.

| ○No, I don't. |
| --- |
| ○Yes, I was diagnosed as _________________.  **(Fill in this item must have been previously diagnosed by the doctor with a psychology-related illness, and cannot be subject to self-perception.)** |
|  |

7. Do you know anything about COVID-19?

| ○I don't know anything about it. |
| --- |
| ○I know a little about it (only know that it is contagious). |
| ○I know some of it (know the transmission route and preventive measures). |
| ○I know much about it (know more about the current epidemic situation, pathogenesis, transmission routes and preventive measures). |
| ○I know very much about it (know the pathogen situation, epidemic characteristics, clinical performance, prevention and control measures, epidemic situation, etc.). |
|  |

8. Have you ever been to Wuhan, Huanggang, Jingmen and other epidemic areas, or have you been in contact with people from Hubei?

| ○No. |
| --- |
| ○Yes. |
|  |

9. Do you have family members, relatives, friends, colleagues and neighbors who have been diagnosed with COVID-19 or suspected patients?

| ○No. |
| --- |
| ○Yes. |
|  |

10. Do you have family, relatives, friends, colleagues, neighbors, etc. around you who are medical staff withstanding COVID-19?

| ○No. |
| --- |
| ○Yes. |
|  |

11. How much do you think this outbreak has affected your daily life and/or work?

| ○Almost nothing. |
| --- |
| ○Some, but the daily life and/or work doesn't need to be changed. |
| ○Much, the daily life and/or work needs to be changed. |
| ○Very much, the daily life and/or work needs to be changed a lot. |

12. Do you often go to some public places after the outbreak-- such as supermarkets, cinemas, parks, libraries, sport places and hotels, etc. (Excluding go out to collect daily necessities and work needs.)

| ○Never. |
| --- |
| ○Sometimes, but less than before the outbreak. |
| ○Same as usual, just like before the outbreak. |
| ○More than usual. |

13. During the Spring Festival holiday, the situation of the epidemic in China is severe. Will you go to visit relatives to have dinner personally?

| ○No, has minimized going out. |
| --- |
| ○Yes, but only to go necessary, significantly less than in previous years. |
| ○Yes, similar to previous years. |
| ○Yes, even more than in previous years. |

14. What measures do you currently take to prevent COVID-19? **(multiple** [**choice**](file:///D:\Dict\7.5.2.0\resultui\dict\?keyword=choice) **question)**

| ○Reducing goes out and avoids contact with people. |
| --- |
| ○Wearing a mask. |
| ○Hand hygiene: washing your hands frequently daily. |
| ○Take Chinese medicine (or other medicine) for prevention. |
| ○Other _________________ |

15. Do you worry about yourself or the people close to you will be infected with COVID-19?

| ○No worry at all. |
| --- |
| ○A little. |
| ○Much. |
| ○Very much. |

16. Do you have the confidence that we can overcome this outbreak?

| ○No confidence at all. |
| --- |
| ○A little，but I don't know when we'll end the outbreak. |
| ○Much，I'm confident that we'll be able to end the outbreak soon. |
| ○Very much, no matter how long it will take to finally overcome the outbreak. |

17. I feel calm . . .

(The following begins with the state anxiety scale (S-AI), which is mainly used for your psychological state after the outbreak.)

| ○Not at all. |
| --- |
| ○Somewhat. |
| ○Moderately so. |
| ○Very much so. |

18. I feel secure . . .

| ○Not at all. |
| --- |
| ○Somewhat. |
| ○Moderately so. |
| ○Very much so. |

19. I'm tense . . .

| ○Not at all. |
| --- |
| ○Somewhat. |
| ○Moderately so. |
| ○Very much so. |

20. I feel strained . . .

| ○Not at all. |
| --- |
| ○Somewhat. |
| ○Moderately so. |
| ○Very much so. |

21. I feel at ease . . .

| ○Not at all. |
| --- |
| ○Somewhat. |
| ○Moderately so. |
| ○Very much so. |

22. I feel upset . . .

| ○Not at all. |
| --- |
| ○Somewhat. |
| ○Moderately so. |
| ○Very much so. |

23. I am presently worrying over possible misfortunes . . .

| ○Not at all. |
| --- |
| ○Somewhat. |
| ○Moderately so. |
| ○Very much so. |

24. I feel satisfied . . .

| ○Not at all. |
| --- |
| ○Somewhat. |
| ○Moderately so. |
| ○Very much so. |

25. I feel frightened. . .

| ○Not at all. |
| --- |
| ○Somewhat. |
| ○Moderately so. |
| ○Very much so. |

26. I feel comfortable . . .

| ○Not at all. |
| --- |
| ○Somewhat. |
| ○Moderately so. |
| ○Very much so. |

27. I feel self-confident . . .

| ○Not at all. |
| --- |
| ○Somewhat. |
| ○Moderately so. |
| ○Very much so. |

28. I feel nervous . . .

| ○Not at all. |
| --- |
| ○Somewhat. |
| ○Moderately so. |
| ○Very much so. |

29. I'm jittery . . .

| ○Not at all. |
| --- |
| ○Somewhat. |
| ○Moderately so. |
| ○Very much so. |

30. I feel indecisive . . .

| ○Not at all. |
| --- |
| ○Somewhat. |
| ○Moderately so. |
| ○Very much so. |

31. I am relaxed . . .

| ○Not at all. |
| --- |
| ○Somewhat. |
| ○Moderately so. |
| ○Very much so. |

32. I feel content. . .

| ○Not at all. |
| --- |
| ○Somewhat. |
| ○Moderately so. |
| ○Very much so. |

33. I am worried. . .

| ○Not at all. |
| --- |
| ○Somewhat. |
| ○Moderately so. |
| ○Very much so. |

34. I feel confused . . .

| ○Not at all. |
| --- |
| ○Somewhat. |
| ○Moderately so. |
| ○Very much so. |

35. I feel steady . . .

| ○Not at all. |
| --- |
| ○Somewhat. |
| ○Moderately so. |
| ○Very much so. |

36. I feel pleasant . . .

| ○Not at all. |
| --- |
| ○Somewhat. |
| ○Moderately so. |
| ○Very much so. |

37. I feel pleasant . . .

(This question begins with the Trait Anxiety Scale (T-AI), which is used to assess your psychological state before the outbreak.)

| ○Almost never. |
| --- |
| ○Sometimes. |
| ○Often. |
| ○Almost always. |

38. I feel nervous and restless . . .

| ○Almost never. |
| --- |
| ○Sometimes. |
| ○Often. |
| ○Almost always. |

39. I feel self-satisfied with myself. . .

| ○Almost never. |
| --- |
| ○Sometimes. |
| ○Often. |
| ○Almost always. |

40. I wish I could be as happy as others seem to be. . .

| ○Almost never. |
| --- |
| ○Sometimes. |
| ○Often. |
| ○Almost always. |

41. I feel like a failure . . .

| ○Almost never. |
| --- |
| ○Sometimes. |
| ○Often. |
| ○Almost always. |

42. I feel rested . . .

| ○Almost never. |
| --- |
| ○Sometimes. |
| ○Often. |
| ○Almost always. |

43. I am “calm, cool, and collected” . . .

| ○Almost never. |
| --- |
| ○Sometimes. |
| ○Often. |
| ○Almost always. |

44. I find that difficulties are piling up so that I cannot overcome them. . .

| ○Almost never. |
| --- |
| ○Sometimes. |
| ○Often. |
| ○Almost always. |

45. I worry too much over something that really don't matter. . .

| ○Almost never. |
| --- |
| ○Sometimes. |
| ○Often. |
| ○Almost always. |

46. I'm happy. . .

| ○Almost never. |
| --- |
| ○Sometimes. |
| ○Often. |
| ○Almost always. |

47. I have disturbing thoughts . . .

| ○Almost never. |
| --- |
| ○Sometimes. |
| ○Often. |
| ○Almost always. |

48. I lack self-confidence . . .

| ○Almost never. |
| --- |
| ○Sometimes. |
| ○Often. |
| ○Almost always. |

49. I feel secure . . .

| ○Almost never. |
| --- |
| ○Sometimes. |
| ○Often. |
| ○Almost always. |

50. I make decisions easily . . .

| ○Almost never. |
| --- |
| ○Sometimes. |
| ○Often. |
| ○Almost always. |

51. I feel inadequate . . .

| ○Almost never. |
| --- |
| ○Sometimes. |
| ○Often. |
| ○Almost always. |

52. I am content . . .

| ○Almost never. |
| --- |
| ○Sometimes. |
| ○Often. |
| ○Almost always. |

53. Some unimportant thought runs through my mind and bothers me . . .

| ○Almost never. |
| --- |
| ○Sometimes. |
| ○Often. |
| ○Almost always. |

54. I take disappointments so keenly that I can’t put them out of my mind . . .

| ○Almost never. |
| --- |
| ○Sometimes. |
| ○Often. |
| ○Almost always. |

55. I am a steady person . . .

| ○Almost never. |
| --- |
| ○Sometimes. |
| ○Often. |
| ○Almost always. |

56. I get in a state of tension or turmoil as I think over my recent concerns and interests . . .

| ○Almost never. |
| --- |
| ○Sometimes. |
| ○Often. |
| ○Almost always. |

57. I feel down-hearted and blue. . .

(This question begins with a Self-rating Depression Scale（SDS）to assess your psychological state after the outbreak.)

| ○A little of the time. |
| --- |
| ○Some of the time. |
| ○Good part of the time. |
| ○Most of the time. |

58. Morning is when I feel the best. . .

| ○A little of the time. |
| --- |
| ○Some of the time. |
| ○Good part of the time. |
| ○Most of the time. |

59. I have crying spells or feel like it. . .

| ○A little of the time. |
| --- |
| ○Some of the time. |
| ○Good part of the time. |
| ○Most of the time. |

60. I have trouble sleeping at night. . .

| ○A little of the time. |
| --- |
| ○Some of the time. |
| ○Good part of the time. |
| ○Most of the time. |

61. I eat as much as I used to. . .

| ○A little of the time. |
| --- |
| ○Some of the time. |
| ○Good part of the time. |
| ○Most of the time. |

62. I still enjoy sex. . .

| ○A little of the time. |
| --- |
| ○Some of the time. |
| ○Good part of the time. |
| ○Most of the time. |

63. I notice that I am losing weight. . .

| ○A little of the time. |
| --- |
| ○Some of the time. |
| ○Good part of the time. |
| ○Most of the time. |

64. I have trouble with constipation. . .

| ○A little of the time. |
| --- |
| ○Some of the time. |
| ○Good part of the time. |
| ○Most of the time. |

65. My heart beats faster than usual. . .

| ○A little of the time. |
| --- |
| ○Some of the time. |
| ○Good part of the time. |
| ○Most of the time. |

66. I get tired for no reason. . .

| ○A little of the time. |
| --- |
| ○Some of the time. |
| ○Good part of the time. |
| ○Most of the time. |

67. My mind is as clear as it used to be. . .

| ○A little of the time. |
| --- |
| ○Some of the time. |
| ○Good part of the time. |
| ○Most of the time. |

68. I find it easy to do the things I used to. . .

| ○A little of the time. |
| --- |
| ○Some of the time. |
| ○Good part of the time. |
| ○Most of the time. |

69. I am restless and can’t keep still. . .

| ○A little of the time. |
| --- |
| ○Some of the time. |
| ○Good part of the time. |
| ○Most of the time. |

70. I feel hopeful about the future. . .

| ○A little of the time. |
| --- |
| ○Some of the time. |
| ○Good part of the time. |
| ○Most of the time. |

71. I am more irritable than usual. . .

| ○A little of the time. |
| --- |
| ○Some of the time. |
| ○Good part of the time. |
| ○Most of the time. |

72. I find it easy to make decisions. . .

| ○A little of the time. |
| --- |
| ○Some of the time. |
| ○Good part of the time. |
| ○Most of the time. |

73. I feel that I am useful and needed. . .

| ○A little of the time. |
| --- |
| ○Some of the time. |
| ○Good part of the time. |
| ○Most of the time. |

74. My life is pretty full. . .

| ○A little of the time. |
| --- |
| ○Some of the time. |
| ○Good part of the time. |
| ○Most of the time. |

75. I feel that others would be better off if I were dead. . .

| ○A little of the time. |
| --- |
| ○Some of the time. |
| ○Good part of the time. |
| ○Most of the time. |

76. I still enjoy the things I used to do. . .

| ○A little of the time. |
| --- |
| ○Some of the time. |
| ○Good part of the time. |
| ○Most of the time. |

Would you like to continue to fill out a new questionnaire, Symptom Checklist 90 (SCL-90). SCL-90 can help you screen for the full psychological impact of the outbreak on you. Your answer is critical to our scientific research, and we urge you to take another 6-8 minutes to complete the last questionnaire.

| ○Yes, I am willing to continue to fill out the follow-up questionnaire. |
| --- |
| ○No, I don't want to continue filling out the follow-up questionnaire. |

77. Please choose the one that best suits you, based on your own situation since the last week.

Followed is a list of problems and complaints that people have. Please read each one carefully. After you have done so, please choose the option which best describes how much that problem has bothered or distressed you during the past 1 week including today. All questionnaires will be treated confidentially!

|  | Not at all | A little bit | Moderately | Quite a bit | Extremely |
| --- | --- | --- | --- | --- | --- |
| Headaches | ○ | ○ | ○ | ○ | ○ |
| Nervousness or shakiness inside | ○ | ○ | ○ | ○ | ○ |
| Unwanted thoughts or ideas that won’t leave your head | ○ | ○ | ○ | ○ | ○ |
| Faintness or dizziness | ○ | ○ | ○ | ○ | ○ |
| Loss of sexual interest or pleasure | ○ | ○ | ○ | ○ | ○ |
| Feeling critical of others | ○ | ○ | ○ | ○ | ○ |
| The idea that someone else can control your thoughts | ○ | ○ | ○ | ○ | ○ |
| Feeling others are to blame for most of your troubles | ○ | ○ | ○ | ○ | ○ |
| Trouble remembering things | ○ | ○ | ○ | ○ | ○ |
| Worried about sloppiness or carelessness | ○ | ○ | ○ | ○ | ○ |

78. Please choose the one that best suits you, based on your own situation since the last week.

|  | Not at all | A little bit | Moderately | Quite a bit | Extremely |
| --- | --- | --- | --- | --- | --- |
| Feeling easily annoyed or irritated | ○ | ○ | ○ | ○ | ○ |
| Pains in heart or chest | ○ | ○ | ○ | ○ | ○ |
| Feeling afraid in open spaces or on the street | ○ | ○ | ○ | ○ | ○ |
| Feeling low in energy or slowed down | ○ | ○ | ○ | ○ | ○ |
| Thoughts of ending life | ○ | ○ | ○ | ○ | ○ |
| Hearing voices that other people do not hear | ○ | ○ | ○ | ○ | ○ |
| Trembling | ○ | ○ | ○ | ○ | ○ |
| Feeling that most people cannot be trusted | ○ | ○ | ○ | ○ | ○ |
| Poor appetite | ○ | ○ | ○ | ○ | ○ |
| Crying easily | ○ | ○ | ○ | ○ | ○ |

79. Please choose the one that best suits you, based on your own situation since the last week.

|  | Not at all | A little bit | Moderately | Quite a bit | Extremely |
| --- | --- | --- | --- | --- | --- |
| Feeling shy or uneasy with the opposite sex | ○ | ○ | ○ | ○ | ○ |
| Feeling of being trapped or caught | ○ | ○ | ○ | ○ | ○ |
| Suddenly scared for no reason | ○ | ○ | ○ | ○ | ○ |
| Temper outbursts that you could not control | ○ | ○ | ○ | ○ | ○ |
| Feeling afraid to go out of your house alone | ○ | ○ | ○ | ○ | ○ |
| Blaming yourself for things | ○ | ○ | ○ | ○ | ○ |
| Pains in lower back | ○ | ○ | ○ | ○ | ○ |
| Feeling blocked in getting things done | ○ | ○ | ○ | ○ | ○ |
| Feeling lonely | ○ | ○ | ○ | ○ | ○ |
| Feeling blue | ○ | ○ | ○ | ○ | ○ |

80. Please choose the one that best suits you, based on your own situation since the last week.

|  | Not at all | A little bit | Moderately | Quite a bit | Extremely |
| --- | --- | --- | --- | --- | --- |
| Worrying too much about things | ○ | ○ | ○ | ○ | ○ |
| Feeling no interest in things | ○ | ○ | ○ | ○ | ○ |
| Feeling fearful | ○ | ○ | ○ | ○ | ○ |
| Your feelings being easily hurt | ○ | ○ | ○ | ○ | ○ |
| Other people being aware of your private thoughts | ○ | ○ | ○ | ○ | ○ |
| Feeling others do not understand you or are unsympathetic | ○ | ○ | ○ | ○ | ○ |
| Feeling that people are unfriendly | ○ | ○ | ○ | ○ | ○ |
| Having to do things very slowly | ○ | ○ | ○ | ○ | ○ |
| Heart pounding or racing | ○ | ○ | ○ | ○ | ○ |
| Nausea or upset stomach | ○ | ○ | ○ | ○ | ○ |

81. Please choose the one that best suits you, based on your own situation since the last week.

|  | Not at all | A little bit | Moderately | Quite a bit | Extremely |
| --- | --- | --- | --- | --- | --- |
| Feeling inferior to others | ○ | ○ | ○ | ○ | ○ |
| Soreness of your muscles | ○ | ○ | ○ | ○ | ○ |
| Feeling that you are watched or talked about by others | ○ | ○ | ○ | ○ | ○ |
| Trouble falling asleep | ○ | ○ | ○ | ○ | ○ |
| Having to check and double check what you do | ○ | ○ | ○ | ○ | ○ |
| Difficulty making decisions | ○ | ○ | ○ | ○ | ○ |
| Feeling afraid to travel on buses, subways or trains | ○ | ○ | ○ | ○ | ○ |
| Trouble getting your breath | ○ | ○ | ○ | ○ | ○ |
| Hot or cold spells | ○ | ○ | ○ | ○ | ○ |
| Having to avoid certain things, places or activities | ○ | ○ | ○ | ○ | ○ |

82. Please choose the one that best suits you, based on your own situation since the last week.

|  | Not at all | A little bit | Moderately | Quite a bit | Extremely |
| --- | --- | --- | --- | --- | --- |
| Your mind going blank | ○ | ○ | ○ | ○ | ○ |
| Numbness or tingling in parts of your body | ○ | ○ | ○ | ○ | ○ |
| A lump in your throat | ○ | ○ | ○ | ○ | ○ |
| Feeling hopeless about the future | ○ | ○ | ○ | ○ | ○ |
| Trouble concentrating | ○ | ○ | ○ | ○ | ○ |
| Feeling weak in parts of your body | ○ | ○ | ○ | ○ | ○ |
| Feeling tense or keyed up | ○ | ○ | ○ | ○ | ○ |
| Heavy feelings in your arms or legs | ○ | ○ | ○ | ○ | ○ |
| Thoughts of death or dying | ○ | ○ | ○ | ○ | ○ |
| Overeating | ○ | ○ | ○ | ○ | ○ |

83. Please choose the one that best suits you, based on your own situation since the last week.

|  | Not at all | A little bit | Moderately | Quite a bit | Extremely |
| --- | --- | --- | --- | --- | --- |
| Feeling uneasy when people are watching or talking about you | ○ | ○ | ○ | ○ | ○ |
| Having thoughts that are not your own | ○ | ○ | ○ | ○ | ○ |
| Having urges to beat, injure or harm someone | ○ | ○ | ○ | ○ | ○ |
| Awakening in the early morning | ○ | ○ | ○ | ○ | ○ |
| Having to repeat the same actions such as touching, counting, washing | ○ | ○ | ○ | ○ | ○ |
| Sleep that is restless or disturbed | ○ | ○ | ○ | ○ | ○ |
| Having urges to break or smash things | ○ | ○ | ○ | ○ | ○ |
| Having ideas or beliefs that others do not share | ○ | ○ | ○ | ○ | ○ |
| Feeling very self-conscious with others | ○ | ○ | ○ | ○ | ○ |
| Feeling uneasy in crowds such as shopping or at a movie | ○ | ○ | ○ | ○ | ○ |

84. Please choose the one that best suits you, based on your own situation since the last week.

|  | Not at all | A little bit | Moderately | Quite a bit | Extremely |
| --- | --- | --- | --- | --- | --- |
| Feeling everything is an effort | ○ | ○ | ○ | ○ | ○ |
| Spells of terror or panic | ○ | ○ | ○ | ○ | ○ |
| Feeling uncomfortable about eating or drinking in public | ○ | ○ | ○ | ○ | ○ |
| Getting into frequent arguments | ○ | ○ | ○ | ○ | ○ |
| Feeling nervous when you are left alone | ○ | ○ | ○ | ○ | ○ |
| Others not giving you proper credit for your achievements | ○ | ○ | ○ | ○ | ○ |
| Feeling lonely even when you are with people | ○ | ○ | ○ | ○ | ○ |
| Feeling so restless you couldn’t sit still | ○ | ○ | ○ | ○ | ○ |
| Feeling of worthlessness | ○ | ○ | ○ | ○ | ○ |
| Feeling that familiar things are strange or unreal | ○ | ○ | ○ | ○ | ○ |

85. Please choose the one that best suits you, based on your own situation since the last week.

|  | Not at all | A little bit | Moderately | Quite a bit | Extremely |
| --- | --- | --- | --- | --- | --- |
| Shouting or throwing things | ○ | ○ | ○ | ○ | ○ |
| Feeling afraid you will faint in public | ○ | ○ | ○ | ○ | ○ |
| Feeling that people will take advantage of you if you let them | ○ | ○ | ○ | ○ | ○ |
| Having thoughts about sex that bother you a lot | ○ | ○ | ○ | ○ | ○ |
| The idea that you should be punished for your sins | ○ | ○ | ○ | ○ | ○ |
| Feeling pushed to get things done | ○ | ○ | ○ | ○ | ○ |
| The idea that something serious is wrong with your body | ○ | ○ | ○ | ○ | ○ |
| Never feeling close to another personal | ○ | ○ | ○ | ○ | ○ |
| Feelings of guilt | ○ | ○ | ○ | ○ | ○ |
| The idea that something is wrong with your mind | ○ | ○ | ○ | ○ | ○ |
