# Supplementary figures and images for: Psychological status and behavior changes of the public during the COVID-19 epidemic in China
Source: Infect Dis Poverty. 2020 May 29;9:58. doi: 10.1186/s40249-020-00678-3 (PMC7256340; doi:10.1186/s40249-020-00678-3)

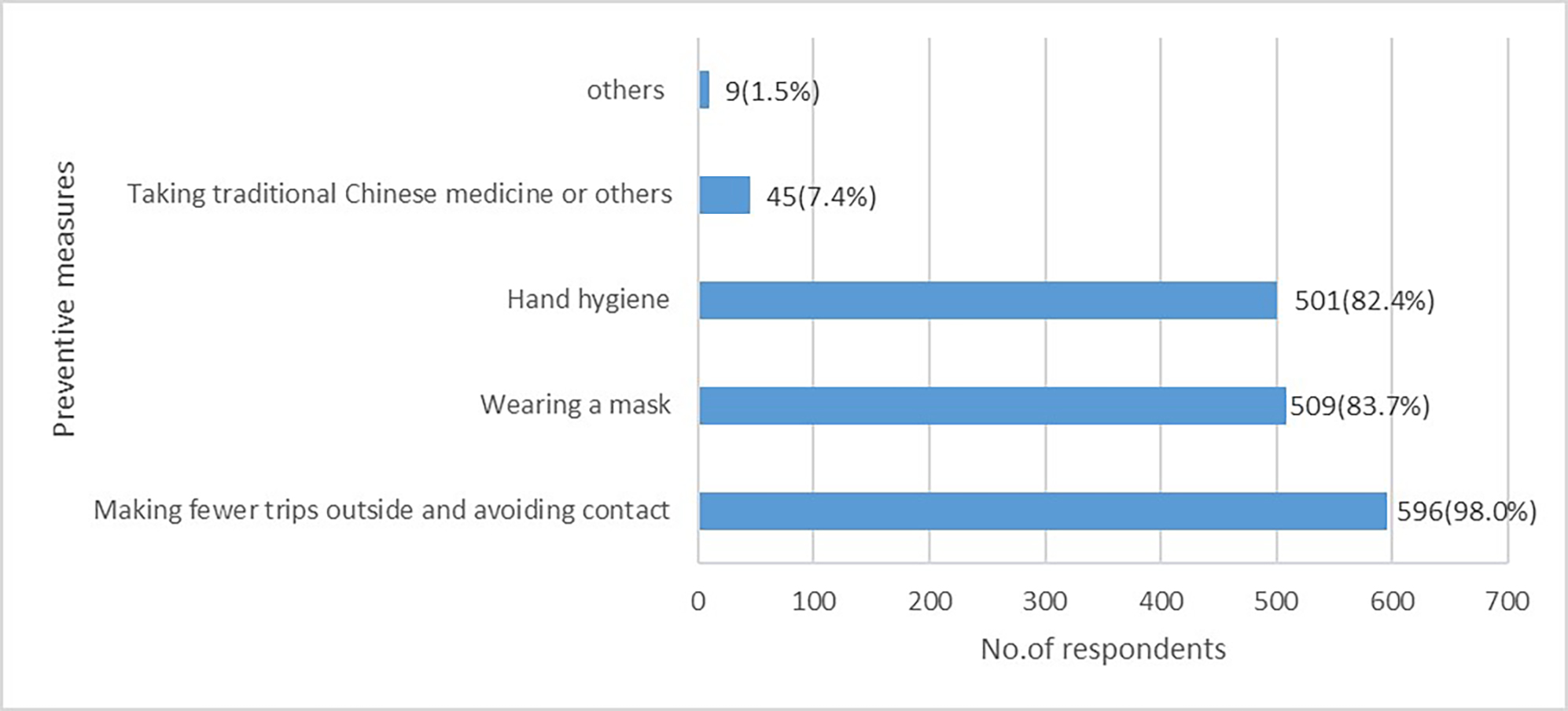

Supplement: Supplementary file 1 — Additional file 1. [file 40249_2020_678_MOESM1_ESM.zip › Supplementary Figure 1 0518.jpg]
